# X-ray crystallographic and high-speed AFM studies of peroxiredoxin 1 from *Chlamydomonas reinhardtii*

**DOI:** 10.1107/S2053230X17018507

**Published:** 2018-01-26

**Authors:** Ratana Charoenwattanasatien, Hideaki Tanaka, Karen Zinzius, Ana K. Hochmal, Risa Mutoh, Daisuke Yamamoto, Michael Hippler, Genji Kurisu

**Affiliations:** a Institute for Protein Research, Osaka University, Suita, Osaka 565-0871, Japan; b Graduate School of Science, Osaka University, Toyonaka, Osaka 560-0043, Japan; c Institute of Plant Biology and Biotechnology, University of Münster, 48143 Münster, Germany; d Faculty of Science, Fukuoka University, Nanakuma, Jyonan-ku, Fukuoka 814-0180, Japan

**Keywords:** photosynthesis, 2-Cys peroxiredoxin, oligomeric state, peroxiredoxin 1, atomic force microscopy, *Chlamydomonas reinhardtii*

## Abstract

Peroxiredoxins (PRXs) are a group of antioxidant enzymes that are found in all organisms, including plants and green algae. The 2-Cys PRX from *Chlamydomonas reinhardtii* (*Cr*PRX1) is a chloroplast-localized protein that is critical for clearing reactive oxygen species in chloroplasts. *Cr*PRX1 is reduced by thioredoxins or calredoxin (*Cr*CRX), a recently identified calcium-dependent redox protein. The molecular interaction between PRXs and thioredoxin/*Cr*CRX is functionally important, but discussion has been limited owing to a lack of structural information on *Cr*PRX1, especially regarding its oligomeric state. In this study, high-speed atomic force microscopy (HS-AFM) images of *Cr*PRX1 and an X-ray crystallographic analysis have enabled examination of the oligomeric state of *Cr*PRX1. Diffraction data from a crystal of the Cys174Ser mutant of *Cr*PRX1 indicate the existence of noncrystallographic fivefold symmetry. HS-AFM images of *Cr*PRX1 further show that *Cr*PRX1 particles form rings with pentagonal rotational symmetry. On the basis of these findings, the oligomeric state of *Cr*PRX1 is discussed and it is concluded that this PRX exists in a ring-shaped decameric form comprising a pentamer of dimers.

## Abstract 2

The oligomeric state of the 2-Cys peroxiredoxin from the green alga *Chlamydomonas reinhardtii* was determined by an X-ray crystallographic study and high-speed AFM image analysis.

## 1. Introduction   

Photosynthesis is a light-dependent chemical process that converts light energy to chemical energy. Light energy is absorbed by chlorophylls or carotenoids in antenna proteins, and is then transferred to photosystem complexes, either photosystem II or I, which perform charge separation at the core of the complex. In nature, the intensity of light perceived by organisms can fluctuate extensively and sometimes exceeds the photosynthetic capacity of the organism. High-intensity light may lead to the production of reactive oxygen species (ROS) such as singlet oxygen (^1^O_2_), superoxide (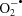), hydroxyl radicals (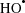) or hydrogen peroxide (H_2_O_2_). These ROS are harmful to photosystems and other chloroplast proteins, and may induce photoinhibition (Takahashi & Badger, 2011 ▸). Plants and other organisms have developed several mechanisms to avoid light-induced photoinhibition, such as ROS-scavenging systems (Asada, 2006 ▸), phototaxis in green alga (Erickson *et al.*, 2015 ▸) and nonphotochemical quenching, which converts the excess light into thermal energy that can be dissipated.

Peroxiredoxins (PRXs) are a group of antioxidant enzymes that are present in all organisms, including plants. In the green alga *Chlamydomonas reinhardtii*, PRXs are classified into four types based on the number and position of cysteine residues: 2-Cys PRX, 1-Cys PRX, PRX-Q and type II PRX (Dayer *et al.*, 2008 ▸). The 2-Cys PRX from *C. reinhardtii* (*Cr*PRX1) is a chloroplast-localized protein that is important for detoxifying ROS in the chloroplast. *Cr*PRX1 can be activated by various types of thioredoxins (TRX; Sevilla *et al.*, 2015 ▸) and its expression is regulated by light and oxygen concentration (Goyer *et al.*, 2002 ▸).

In general, the oligomeric state of 2-Cys PRX is a dimer or a decamer, depending on the redox state, protein concentration, pH or its physiological function (Barranco-Medina *et al.*, 2009 ▸). In the crystal structure, the human homologues of 2-Cys PRX, peroxiredoxin 3 (PRX3; PDB entry 5jcg) and peroxi­redoxin 4 (PRX4; PDB entry 3tkp), form decamers (Yewdall *et al.*, 2016 ▸; Wang *et al.*, 2012 ▸). The cryo-TEM structure of PRX3 shows that decameric ring structures are stacked into a long helical filament, which might function as a self-associating chaperone. The crystal structure of 2-Cys PRX from the bacterium *Salmonella typhimurium* shows that the oxidized enzyme is in the decameric state, and its signalling activity is regulated by a redox-sensitive state change from a dimer to a decamer (Wood *et al.*, 2002 ▸). Regarding plants, the crystal structure of PRX (type II) from *Populus trichocapa* has been reported (Echalier *et al.*, 2005 ▸). This plant PRX forms a dimer in both the crystal (PDB entry 1tp9) and in solution, as confirmed by X-ray crystallography and NMR spectroscopy, respectively. Thus, the oligomerization of PRXs is functionally important, but is very difficult to predict based only on amino-acid sequence information.

Recently, we found that *Cr*PRX1 is activated not only by TRXs but also by calredoxin (*Cr*CRX), a novel chloroplast-localized calcium-dependent protein consisting of calmodulin and thioredoxin domains (Hochmal *et al.*, 2016 ▸). A possible mode of interaction between *Cr*PRX1 and *Cr*CRX was proposed on the basis of the crystal structure of *Cr*CRX (PDB entry 5e37), but the preliminary discussion was limited owing to a lack of structural information on *Cr*PRX1, especially on its oligomeric state. Because *C. reinhardtii* is a model organism in photosynthesis research, structural information on *Cr*PRX1 would be useful for further analysis of photoacclimation in plant physiology or plant biology, as well as photosynthesis research, and is particularly important for the understanding of Ca^2+^-dependent electron transfer between *Cr*PRX1 and *Cr*CRX. The aim of the present study was therefore to analyze a single-particle image of recombinant *Cr*PRX1 using high-speed atomic force microscopy (HS-AFM) and to crystallize *Cr*PRX1 for further structural analysis. Based on the low-resolution HS-AFM image of *Cr*PRX1 and the crystallo­graphic analysis described here, we discuss the oligomeric state of *Cr*PRX1.

## 2. Materials and methods   

### 2.1. Cloning, protein expression and purification, and biochemical estimation of molecular weight   

**Figure 1:**
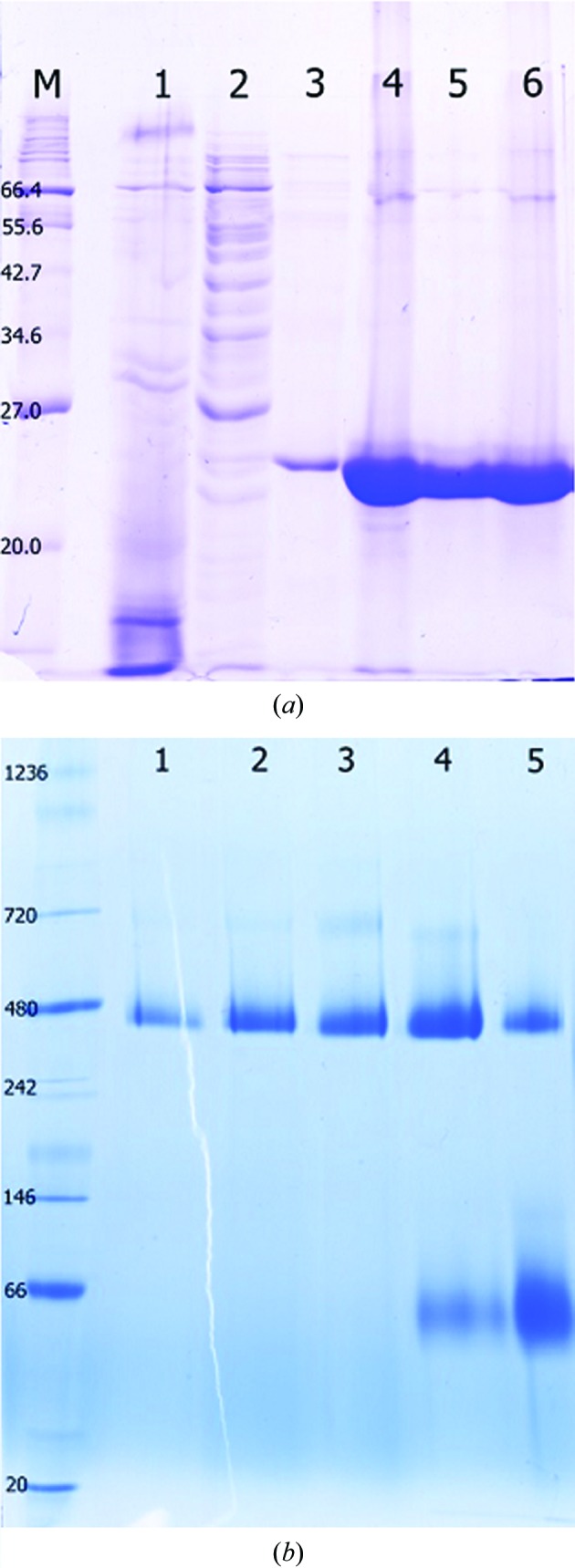
SDS–PAGE and native PAGE analysis of purified wild-type *Cr*PRX1 protein and its crystals. (*a*) SDS–PAGE showing the purification of *Cr*PRX1 by Ni–NTA and gel-filtration chromatography. Lane 1, flowthrough from the nickel column. Lanes 2 and 3, fractions from the 20 m*M* imidazole wash. Lane 4, elution of *Cr*PRX1 with 250 m*M* imidazole. Lanes 5 and 6, fractions from Superdex 200. (*b*) Native PAGE of *Cr*PRX1 from dissolved crystals and solution samples. Lanes 1–3, *Cr*PRX1 crystals. Lanes 4 and 5, *Cr*PRX1 from stock protein solution.

The plasmid for the expression of recombinant *C. reinhardtii* peroxiredoxin 1 (*Cr*PRX1) was constructed as described previously (Hochmal *et al.*, 2016 ▸) and summarized in Table 1 ▸. The pET-22b *Cr*PRX1 plasmid was transformed into *Escherichia coli* strain BL21(DE3). A single Cys174Ser (C2S) mutant of *Cr*PRX1, which is a mutant that mimics the reduced active-site cysteine residues, was engineered in the pET-22b *Cr*PRX1 plasmid by an In-Fusion Cloning kit (Takara Bio USA, Mountain View, California, USA) using the primers 5′-GAGGTCAGCCCCGCCGGCTGGAAG-3′ and 5′-GGCGGGGCTGACCTCATCGGGGTT-3′. Wild-type and C2S mutant cells were grown in LB medium with 100 µg ml^−1^ ampicillin and incubated at 310 K with shaking until the OD_600_ reached 0.5–0.6. Protein expression was induced with 0.5 m*M* isopropyl β-d-1-thiogalactopyranoside, and the cells were further incubated at 310 K for 4–6 h. The cells were harvested by centrifugation at 8000*g* for 10 min and stored at 193 K. The cell pellets were suspended in lysis buffer consisting of 50 m*M* Tris–HCl pH 8.0, 150 m*M* NaCl, 1 m*M* phenylmethylsulfonylfluoride and disrupted by sonication. The lysate was clarified by ultracentrifugation at 164 430*g* for 30 min and the supernatant was applied onto Ni-NTA resin (Qiagen, Hilden, Germany) pre-equilibrated with 50 m*M* Tris–HCl pH 8.0, 150 m*M* NaCl. The Ni-NTA resin was washed and eluted with 20 and 250 m*M* imidazole in equilibration buffer. The *Cr*PRX1 protein was further purified using gel filtration on a Superdex 200 HR 16/60 column (GE Healthcare). The column was equilibrated with 20 m*M* HEPES pH 7.5, 150 m*M* NaCl. Purified protein fractions were concentrated by centrifugation using Amicon filter units with a membrane nominal molecular-weight limit of 10 kDa (Merck Millipore, Cork, Ireland) for crystallization. Purified protein fractions were concentrated and kept at 193 K for crystallization (Fig. 1*a* ▸).

The molecular weight of wild-type *Cr*PRX1 was first estimated using a gel-filtration column with several protein markers. To verify the estimated molecular weight of *Cr*PRX1 both in solution and from dissolved crystal samples, 20 µg protein sample or several crystals of *Cr*PRX1 were loaded onto gels for SDS–PAGE and native PAGE (NativePAGE Novex 4–16% Bis-Tris Gels) analysis (Fig. 1 ▸). Macromolecule-production information is summarized in Table 1 ▸.

### 2.2. Crystallization   

**Figure 2:**
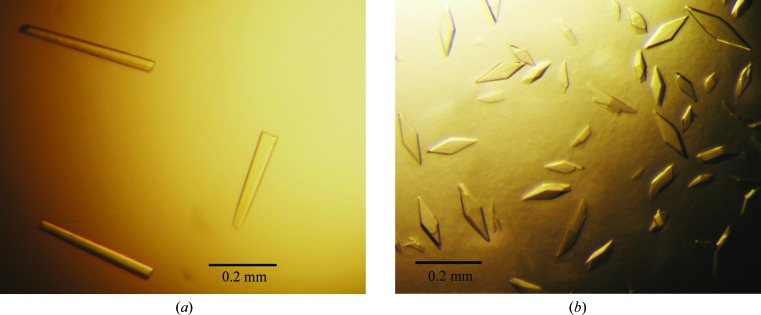
Photographs of typical crystals of wild-type *Cr*PRX1 and the C2S mutant. (*a*) Crystals of wild-type *Cr*PRX1. Crystals were obtained in a droplet consisting of 0.1 *M* sodium citrate pH 7.5, 50 m*M* ammonium acetate, 21% polyethylene glycol 400, 15% glycerol at 277 K. (*b*) Crystals of the C2S mutant of *Cr*PRX1. Crystals were obtained in a droplet consisting of 0.1 *M* HEPES pH 7.6, 0.2 *M* sodium thiocyanate, 20% PEG 3350 at 277 K.

Crystallization conditions for *Cr*PRX1 were screened by the hanging-drop vapour-diffusion method using Crystal Screen, Crystal Screen 2, PEG Rx 1, PEG Rx 2 (Hampton Research, Aliso Viejo, California, USA) and Wizard I, II, III and IV (Rigaku, Bainbridge Island, Washington, USA) at 277 and 293 K. Needle crystals of wild-type *Cr*PRX1 were produced using 0.2 *M* ammonium acetate, 0.1 *M* sodium citrate pH 5.5, 24% PEG 400. After optimization including a microseeding technique, crystals with a suitable size for an X-ray experiment were obtained from droplets comprised of 1 µl of 30 mg ml^−1^ protein sample and 1 µl reservoir solution consisting of 0.05 *M* ammonium acetate, 0.1 *M* sodium citrate pH 5.5, 20% PEG 400, 15% glycerol (Fig. 2 ▸ *a*). Unexpectedly, the crystallization conditions for the C2S mutant of *Cr*PRX1 differed markedly from those for the wild type. After optimization of the crystallization conditions, rhombic crystals of the C2S mutant were obtained from droplets comprised of 2 µl of 15 mg ml^−1^ protein sample and 1 µl reservoir solution consisting of 0.1 *M* HEPES pH 7.6, 0.2 *M* sodium thiocyanate, 20% PEG 3350 (Fig. 2 ▸ *b*). Crystallization information is summarized in Table 2 ▸.

### 2.3. X-ray data collection and processing   

Single wild-type *Cr*PRX1 crystals were picked up and directly cooled in liquid nitrogen. Single crystals of the C2S mutant were picked up and first soaked in 20% glycerol for cryoprotection before being cooled in liquid nitrogen. X-ray diffraction images were collected from single crystals of wild-type and C2S mutant *Cr*PRX1 on the BL44XU beamline at SPring-8. Data sets for wild-type *Cr*PRX1 were collected at 100 K using a CCD-based MX300-HE detector system with an oscillation angle of 0.5° per frame, a crystal-to-detector distance of 450 mm and an exposure time of 1 s per frame. Data sets for C2S mutant *Cr*PRX1 were collected at 100 K using the same CCD-based detector. The oscillation angle per frame was 0.3° and the crystal-to-detector distance was 350 mm. The exposure time per frame was 1 s. The diffraction data for the wild type were processed with the *HKL*-2000 software package (Otwinowski & Minor, 1997 ▸) and the data for the C2S mutant were processed with the *XDS* program package (Kabsch, 2010 ▸). Data-collection and processing statistics are summarized in Table 3 ▸.

### 2.4. High-speed atomic force microscopy (HS-AFM)   

The concentration of *Cr*PRX1 was 0.1 mg ml^−1^ in imaging buffer (50 m*M* sodium acetate pH 5.5, 150 m*M* NaCl). 2 µl of 100 m*M* CoCl_2_ was loaded onto freshly cleaved mica substrate for 3 min at room temperature. After washing the mica surface with imaging buffer, 2 µl of *Cr*PRX1 was loaded onto the mica surface and then incubated for 5 min at room temperature. After washing the mica surface with imaging buffer, HS-AFM imaging was performed in amplitude-modulation mode using a laboratory-built HS-AFM setup. The cantilever used was BL-AC10DS-A2 (Olympus Co., Tokyo, Japan) with a tip fabricated by electron-beam deposition.

## 3. Results and discussion   

Recombinant *Cr*PRX1 protein comprising 221 amino acids was successfully overexpressed and purified to electrophoretic homogeneity, showing a molecular weight of 24.7 kDa on SDS–PAGE (Fig. 1 ▸ *a*). Native PAGE and gel-filtration chromatographic analysis showed that *Cr*PRX1 exists as an oligomer in solution (Fig. 1 ▸ *b*). The molecular mass of *Cr*PRX1 from both dissolved crystals and purified solution was estimated to be about 350–480 kDa, indicating a clearly greater molecular mass than that of the homodimer, in contrast to the oligomeric state of type II PRX1 from higher plants (Echalier *et al.*, 2005 ▸). However, the solution sample eluted from gel-filtration column chromatography (lanes 4 and 5 in Fig. 1 ▸ *b*) also contained a band at about 66 kDa corresponding to the dimeric form. This observation suggests that *Cr*PRX1 can adopt both dimeric and higher oligomeric forms but prefers the oligomeric form, which can be crystallized.

**Figure 3:**
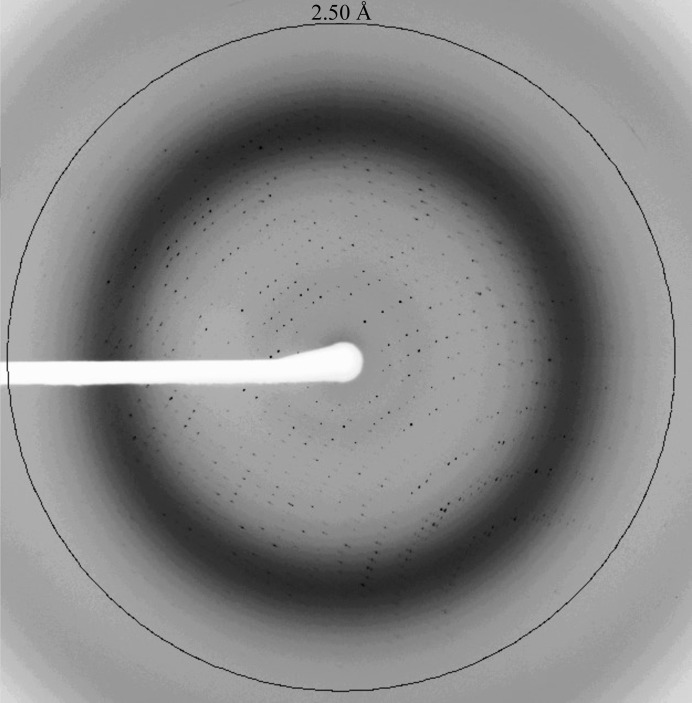
Diffraction image of a *Cr*PRX1 crystal of the C2S mutant recorded on BL44XU at SPring-8.

**Figure 4:**
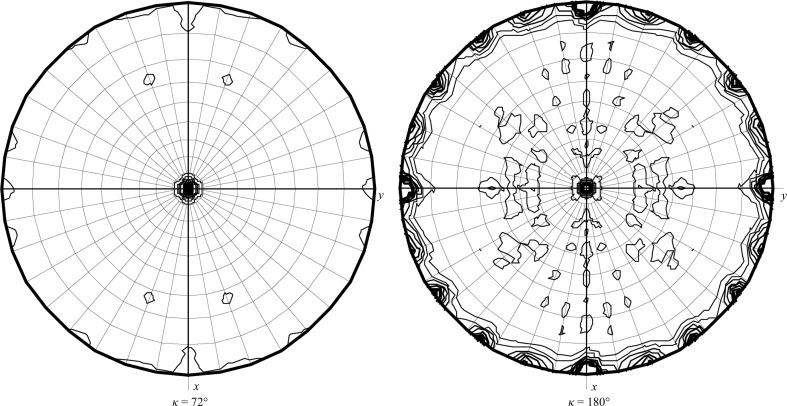
Self-rotation functions of the C2S mutant of *Cr*PRX1 at κ = 72° and 180°. Strong peaks are also found every 72°. The peak heights at the κ = 72, 144, 180, 216, 288 and 360° sections are 119 700, 160 200, 302 200, 160 200, 119 700 and 302 200, respectively.

The crystal of wild-type *Cr*PRX1 was found to belong to the hexagonal space group *P*6_3_, with unit-cell parameters *a* = *b* = 137.12, *c* = 354.87 Å, but only diffracted to 4.8 Å resolution (Table 2 ▸). In contrast, the crystal of the C2S mutant was found to belong to space group *C*222_1_, with unit-cell parameters *a* = 134.81, *b* = 418.11, *c* = 94.32 Å, and diffracted to 2.4 Å resolution, thus being suitable for atomic structure analysis (Fig. 3 ▸). This improvement in crystal quality may be owing to the different packing of the C2S mutant crystals. For phasing, we carried out molecular replacement using the program *Phaser MR* in the *CCP*4 program suite (Collaborative Computational Project, Number 4, 1994 ▸; Winn *et al.*, 2011 ▸). The best homologous model for the molecular-replacement calculations was thioredoxin peroxidase B from human red blood cells (PDB entry 1qmv; Schröder *et al.*, 2000 ▸), with 63% sequence identity to *Cr*PRX1. Structure determination, including model rebuilding and refinement, is now in progress, but we note that the self-rotation functions calculated using the diffraction data from the C2S crystal show an obvious peak in the κ = 72° section corresponding to the existence of noncrystallographic fivefold symmetry (Fig. 4 ▸).

**Figure 5:**
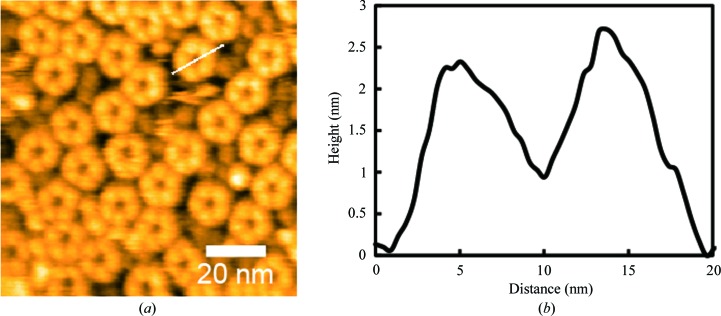
HS-AFM images of *Cr*PRX1. (*a*) Topography image. (*b*) Height profile of a *Cr*PRX1 particle along the white line in (*a*). The imaging rate was 1 s per frame.

Importantly, HS-AFM images revealed that the wild-type *Cr*PRX1 particles formed rings with pentagonal rotational symmetry in solution (Fig. 5 ▸). This corresponds well to the fivefold symmetry found in the self-rotation function described above, illustrating that the noncrystallographic fivefold symmetry is intramolecular. The average peak height of AFM images from the mica surface was 2.7 ± 0.3 nm (*n* = 30). The observed outer diameter of the *Cr*PRX1 ring was 16.8 ± 0.94 nm (*n* = 30) on average. Together with the observed fivefold symmetry and the experimentally measured height and diameter of *Cr*PRX1, the possibility of a highly stacked ring structure, as found in the filamentous oligomeric state of PRX3 (Yewdall *et al.*, 2016 ▸), is unlikely. Although the only available structure of a plant PRX (that from *P. trichocapa*) is homodimeric (PDB entry 2pwj; Barranco-Medina *et al.*, 2006 ▸), the search model for molecular replacement with the highest sequence identity (PDB entry 1qmv) possessed a decameric ring structure. Considering other examples of 2-Cys PRXs, such as PRX3, PRX4 and PRX5, we conclude that the oligomeric state of *Cr*PRX1 is a ring-shaped decamer formed by a pentamer of dimers both in the crystal and solution states. Efforts towards structure determination using the molecular-replacement method are currently progressing well, and will provide a clearer insight into the oligomeric state of *Cr*PRX1, as well as being essential for elucidating the functional interaction with TRXs and *Cr*CRX.

## Supplementary Material

A gel-filtration profile.. DOI: 10.1107/S2053230X17018507/nw5068sup1.pdf

## Figures and Tables

**Table 1:** Macromolecule-production information

Source organism	*C. reinhardtii*
DNA source	RNA from *C. reinhardtii*
Forward primer	5′-GGTATTCCATATGGCTTCCCACGCCGAG-3′
Reverse primer	5′-CGGGATCCTGCACGGCAGAGAAGTACTCC-3′
Cloning vector	pET-22b(+) (Novagen)
Expression vector	pET-22b(+) (Novagen)
Expression host	*E. coli* strain BL21(DE3)
Complete amino-acid sequence of the construct produced	MASHAEKPLVGSVAPDFKAQAVFDQEFQEITLSKYRGKYVVLFFYPLDFTFVCPTEITAFSDRYKEFKDINTEVLGVSVDSQFTHLAWIQTDRKEGGLGDLAYPLVADLKKEISKAYGVLTEDGISLRGLFIIDKEGVVQHATINNLAFGRSVDETKRVLQAIQYVQSNPDEVCPAGWKPGDKTMKPDPKGSKEYFSAVQDPNSSSVDKLAAALEHHHHHH

**Table 2:** Crystallization

	Wild type	C2S mutant
Method	Hanging-drop vapour diffusion	Hanging-drop vapour diffusion
Plate type	Hampton Research 48-well	Hampton Research 48-well
Temperature (K)	277	277
Protein concentration (mg ml^−1^)	30	15
Buffer composition of protein solution	20 m*M* HEPES pH 7.5, 150 m*M* NaCl	20 m*M* HEPES pH 7.5, 150 m*M* NaCl
Composition of reservoir solution	0.05 *M* ammonium acetate, 0.1 *M* sodium citrate pH 5.5, 20% PEG 400, 15% glycerol	0.2 *M* sodium thiocyanate, 0.1 *M* HEPES pH 7.6, 20% PEG 3350
Volume and ratio of drop	1 µl + 1 µl	2 µl + 1 µl
Volume of reservoir (µl)	150	150

**Table 3:** Data collection and processing Values in parentheses are for the outer shell.

	Wild type	C2S mutant
Diffraction source	BL44XU, SPring-8	BL44XU, SPring-8
Wavelength (Å)	0.90000	0.90000
Temperature (K)	100	100
Detector	Rayonix MX300-HE	Rayonix MX300-HE
Crystal-to-detector distance (mm)	450	350
Rotation range per image (°)	0.5	0.3
Total rotation range (°)	120	90
Exposure time per image (s)	5	1
Space group	*P*6_3_	*C*222_1_
*a*, *b*, *c* (Å)	137.13, 137.13, 354.87	134.81, 418.11, 94.32
α, β, γ (°)	90, 90, 120	90, 90, 90
Mosaicity (°)	0.616–0.820	0.197
Resolution range (Å)	50–4.80 (4.97–4.80)	47.37–2.40 (2.45–2.40)
Total No. of reflections	131875	381061
No. of unique reflections	18138	194153
Completeness (%)	99.7 (99.9)	96.4 (98.8)
Multiplicity	7.3 (7.1)	1.96 (1.96)
〈*I*/σ(*I*)〉	15.3 (6.6)	4.65 (1.95)
*R*_merge_ (%)	13.6 (40.7)	9.8 (58.4)
Overall *B* factor from Wilson plot (Å^2^)	34.797	52.6
