# Supplementary material for: X-ray crystallographic and high-speed AFM studies of peroxiredoxin 1 from Chlamydomonas reinhardtii
Source: Acta Crystallogr F Struct Biol Commun. 2018 Jan 26;74(Pt 2):86–91. doi: 10.1107/S2053230X17018507 (PMC5947678; doi:10.1107/S2053230X17018507)
Supplement: Supplementary file 1 [file f-74-00086-sup1.pdf]

STRUCTURAL BIOLOGY
COMMUNICATIONS

Volume 74 (2018)

Supporting information for article:

X-ray crystallographic and high-speed AFM studies of peroxiredoxin-1 from *Chlamydomonas reinhardtii*

Ratana Charoenwattanasatien, Hideaki Tanaka, Karen Zinzius, Ana K. Hochmal, Risa Mutoh, Daisuke Yamamoto, Michael Hippler and Genji Kurisu

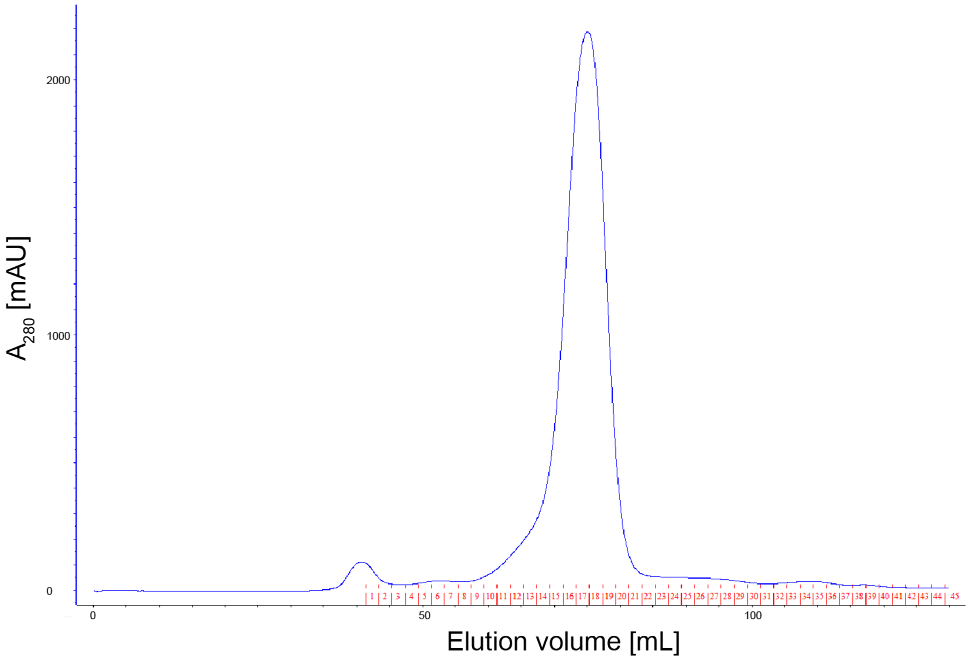

Supplementary Figure S1: A gel-filtration profile of wild-type *Cr*PRX1.
